# Assessment of Pharmacists Experiences and Attitudes Toward Professionalism and its Challenges in Pharmacy Practice

**Published:** 2018

**Authors:** Mohammadreza Javadi, Nikinaz Ashrafi, Pooneh Salari

**Affiliations:** a *Department of Clinical Pharmacy, Faculty of Pharmacy, Tehran University of Medical Sciences. *; b *Faculty of Pharmacy, Tehran University of Medical Sciences, Tehran, Iran. *; c *Medical Ethics and History of Medicine Research Center, Tehran University of Medical Sciences.*

**Keywords:** Pharmacy ethics, Ethical professional practice, Pharmacy practice, Professionalism, Professional ethics

## Abstract

Nowadays pharmacists should be involved in patients care and providing pharmaceutical care more than before, but still there is a gap between standard of care and pharmacy practice in pharmacies. In this study we aimed at evaluating the pharmacists experiences and attitudes about ethical professional practice in pharmacies. The study was conducted in the Tehran University of Medical Sciences, Tehran, Iran.

This study performed as a mixed method study including 12 semi-structured interviews and two focus group discussions (FGDs). All interviews and FGDs were recorded verbatim. The study evaluates the pharmacy practice based on the Code of Ethics for National Pharmaceutical System requirements.

Our study presents the pharmacists ethical challenges in 14 areas of practice such as lack of proper pharmacists-patients and inter and intra-professional relationship; poor management of medication error; lack of pharmacists awareness about their responsibilities, professional rules and regulations; non-OTC drug dispensing without prescription; no collaboration with custodian organizations; dissatisfaction from profession; financial problems; mismanagement in confronting with ads and offers of pharmaceutical companies, and conflict of interest; and uneven drug distribution during shortage.

For providing standard pharmaceutical care modification of infra structures, educational system and regulations in pharmaceutical system is highly recommended.

## Introduction

Nowadays the role of pharmacists has changed from drug dispensing to providing patient-centered pharmaceutical care; so they should be involved in patients follow-ups, promoting patients compliance, preventing adverse drug reactions and interactions. In addition, it has been suggested that pharmacists may involve in public health programs such as immunization, lifestyle modification, management of chronic long lasting diseases (such as diabetes), in order to improve overall health status (1). The accreditation standards from the Accreditation Council for Pharmacy Education (ACPE) indicates that pharmacy graduates should be able to “promote health and wellness and describe the influence of population-based care on patient-centered care” and “identify problems, explore and prioritize potential strategies (2). Concerning these activities and the advancements in pharmaceutical care services, more emphasis should be made on professionalism in pharmacy practice and inclusion of pharmacy ethics in pharmacy education. In fact integrating professionalism and pharmacy ethics into practice and empowering pharmacy education from scientific point of view, guarantees the quality of pharmaceutical care services. Concerning pharmacy ethics education, from about 10 years ago pharmacy ethics is taught in faculties of pharmacy of Iran as one unit course which does not seem to sufficiently cover the required topics. Also a Code of Ethics for National Pharmaceutical System was compiled in 2013 which indicates the ethical perspectives of professional practice and the principle values of the pharmaceutical system (3,4), but still there seems to be a gap between ethical professional considerations and pharmacists deliberation and action. In this regard and because the PharmD graduates are mostly facing with ethical and professional challenges while provide pharmaceutical services in pharmacies, we focused at evaluating the pharmacists experiences of professionalism and its challenges in community pharmacies. Hopefully our study will present the real gap which lights on modification of infrastructures and pharmacy educational system. 

## Methods

In this study we evaluated the pharmacists’ experiences in a mixed method study, deep interviews, and focus group discussions.

*First phase*

*Participants*

Pharmacists, who were working in community pharmacies or governmental pharmacies were purposively recruited to represent their different experiences and ethical and professional challenges which they are faced with in their routine practice. The recruited pharmacists from community pharmacies were technical managers practicing in private pharmacies or technical manager and stakeholder. They were called through the registration system of the Fourth Annual Meeting of Clinical Pharmacists and continuing medical education (CME) program using a passive snowballing technique to simplify recruitment. Some of the technical managers were recruited from governmental pharmacies related to the Tehran University of Medical Sciences, 13 Aban Pharmacy (affiliated with Tehran University of Medical Sciences). 

*Procedure*

An interview guide was generated using the Code of Ethics for National Pharmaceutical System ^3^ to seek the pharmacists’ experiences and challenges around the issue. This guide consisted of 10 questions based on key principles of the mentioned code. As the code was composed of the principles of bioethics and professionalism, 10 questions considering the 8 articles of the code including respects for patients dignity and autonomy, beneficence, non-maleficence, justice, empathy, honesty, cooperation and excellence were designed. At the beginning, all participants were informed about the study and ensured about their confidentiality and voluntariness; and finally they gave their verbal consent for participation. All of the participants were taken part in a semi-structured interview using the theme guide. All interviews were audio recorded. The study continued until data saturation.

*Data analysis*

The recorded interviews were transcribed verbatim more than three times to get familiarized with the qualitative data through frequent readings and note takings. The initial themes were generated, defined and structured in the provision of the main themes of the Code of Ethics for National Pharmaceutical System. 

*Second phase*

For performing the second phase of the study, two focus group discussions (FGD) were generated. The first FGD consisted of the last year residents of clinical pharmacy who were familiar with pharmacy practice and pharmaceutical care. In the second FGD the PhD graduates in pharmaceutical sciences or the last year students of pharmaceutical sciences PhD programs participated. In order to maximize disclosure among FGD participants, homogeneity was considered in each FGD. All of the participants were nominated by one of the researchers. 

*Procedure*

The results of the phase one, provided a theme guide for the next two FGDs. All participants were informed about the study and ensured about their confidentiality and voluntariness at the beginning of each FGD; and finally they gave their verbal consent for participation. The FGDs were recorded. 

*Data analysis*

The recorded FGDs were transcribed verbatim through frequent readings and note takings. The FGDs findings were organized in the synthesized format.

*Ethical approval*

The study was approved by the Ethics Committee of the Tehran University of Medical Sciences. All participants provided verbal consent. 

## Results

*First phase*

 Twelve face to face deep interviews were performed. Of interviewees six pharmacists were stake holder and technical manager; three were technical manager in community pharmacies; three were technical manager in government/institutional pharmacies. The participants’ demographic data is summarized in Table 1. Sample data generated from interviews are presented as findings in italics to represent participants experiences. The main themes that resulted from the analysis were lack of proper pharmacists-patients relationship, poor management of medication error, non-OTC drug dispensing without prescription, lack of inter and intra professional relationship, lack of pharmacists awareness about their responsibilities, no collaboration with custodian organizations, lack of awareness about professional rules and regulations, lack of proper support for internal productions, dissatisfaction from profession, financial problems (lack of professional relationship between technical manager and stakeholder, mismanagement in confronting with ads and offers of pharmaceutical companies, selling supplements and herbal products), no official retirement plan based on age, Uneven drug distribution during shortage, and conflict of interest. All data were presented in Table 2. 

 Economy and financial benefits were considered as the most effective determinants of the quality of professional health care services. Of twelve interviews, it is suggested that economic and financial issues similarly affects pharmacy practice. The following quotes highlight the pharmacists point of view about financial and economic issue:

“Because the pharmacy is business organization, if the physicians would like to not to have collaboration with pharmacists (sending the patients to our pharmacies) the pharmacist should negotiate and sometimes should violate ethical principles.”

“If I finance the pharmacy by myself (the technical manager and the stakeholder are the same), mostly I have to be concerned about economic”

Unplanned pharmacy practice in confronting with conflict of interest, medication error, inter- and intra-professional relationships, no consciousness about relationship with patients, and drug shortages seem to be as a consequence of lack of pharmacists awareness about their responsibility and the rules and regulations of their profession. Furthermore insecure relationship with patients was dependent on the physical limitations of the pharmacy environment and time shortage. The following phrases illustrate these issues:

“Because of our relationship with pharmaceutical companies, when our relationship is more friendly we allow them to make propaganda for their products even if we know that their advertisements are not scientifically accepted.”

“If I confront with medication error especially error in drug order, firstly I call the physician. If I make sure about the error and the physician does not have proper cooperation and does not accept his error, I solve the problem by myself, otherwise I refer the patient to another physician.”

“Sometimes the physician asks us not to inform the patient about the dosage; afterwards he asks the patient to go back to the physicians office and then the secretary of the physician indicates the dosage.” 

“We as the pharmacists do not support and accept each other”

“In relationship with patients the time and financial issues are important, previously our profit was more than now”

“Pharmacists patients relationship wastes patients time and negatively affects our financial profit.”

“I do drug rationing when I am confronting drug shortage and I am trying to give lesser drugs to more patients.”

 Only after entering into pharmacy practice the pharmacists get learned about rules and regulations by trial and error and unfortunately there is no proper professional support on behalf of custodian organizations or pharmaceutical companies. The participants explained their view as below:

“Newly graduated pharmacists are not familiar with the rules and regulations and there is no defined reference to guide us.”

*Second phase*

 At this phase two FGDs were formed, the first one by participation of seven clinical pharmacist residents and the second one was consisted of six pharmaceutics specialists. The demographic data of participants was presented in Table 3. We considered the clinical pharmacy residents as the skilled clinical practitioners in pharmacy who are spending most of their times in hospitals and pharmacies. Also pharmaceutics specialists were participated in the second FGDs as specialists whose practice is directly related to the industries, because the industries are considered as the strength of every pharmaceutical system. 

 Our results show that all of the participants confirmed the data generated in the first phase, discussed about each of them in order to find a solution. Their point of view and recommendations are illustrated in Table 4. They provided an insight into solving the problem and implication of professionalism and pharmacy ethics. Their recommendation can be assorted into 5 categories including knowledge development, skill advancement, service provision, regulation adjustment and management and audit. The participants of the first FGD mostly insisted on modifications in educational system, skill advancement, regulation adjustment and service provision while the results of the second FGD set the goal at modification of educational system and regulatory bodies. 

## Discussion

Our study shows that there is significant gap between the standard of pharmaceutical care and current pharmacy practice in our pharmacies. Although our study results determine financial problems as the most affecting issue on pharmacy practice, it is suggested that the Iranian pharmacists are not familiar with their ethical professional responsibilities. In agreement with these results, our former studies showed that the pharmacists attitude toward ethics and professionalism is at the medium and low level (5, 6).

The Code of Ethics for National Pharmaceutical System precisely reminds the ethical aspects and the principle values of professional pharmacy practice. Professional ethics education, professional codes and the method of implicating moral judgement help ethical professional practice. In fact professional ethics education empowers the professionals to analyze the ethical dilemma, interpret the situation and make ethical decision (7) which should be based on relevant principles. 

For all health care providers, collaborative team work with other disciplines is considered as a necessary skill but there are some obstacles limiting proper inter and intra-professional collaboration including cultural differences between disciplines, the process of decision making, and professionals perception of the teamwork (8-10). Our study shows lack of trust and existence of misconceptions between inter and intra-professionals as the barriers. According to the report of the Institute of Medicine (IOM), no appropriate inter and intra-professional relationship is considered as one of the leading problems in U.S. health care system. Based on this report the health care professionals prefer practicing alone instead of being as a patient-centered team (11). Inter-professional Education Collaborative (IPEC) brought up the Core Competencies for Inter-professional Collaborative Practice in 2009 (8). The mentioned core competencies are categorized into four domains including values/ethics, roles/responsibilities, inter-professional communication, and team and teamwork. The 2010 World Health Organization report insisted on inter and intra-professional relationship and presents the “collaborative practice-ready health workforce” as a universal goal for inter-professional education (IPE) which helps effective collaboration and improves quality of care and its consequences (12). Also the participants of our study indicated non-professional behavior and lack of professional integrity as the consequences of lack of inter and intra-professional relationship. So the necessity of IPE is highly feeling and all of the core competencies should be included. Luetsch *et al.* addressed lack of confidence in beginning inter-professional communication by pharmacists and suggested teaching communication skills as a solution to improve their perceived capability and confidence in intra-professional relationship (13). In a study conducted by West *et al*., it was showed that one IPE activity or curricular element is not adequate and the IPEC competencies should be more comprehensively addressed in curriculum (9). Seselja-Perisin *et al.*, indicated the negative impact of students lack of openness and positive attitude toward inter-professional collaboration on inter-professional environment which results in improper patients care. They assumed improvement in collaboration by modification of the undergraduate curriculums (14). 

Failure by a pharmacist especially a hospital pharmacist to have effective communication with patients may negatively affect patients adherence and compliance to medication and impair quality of health care services (15, 16). The ACPE standards declares that introductory pharmacy practice experiences (IPPEs) should start soon in the curriculum in association with classroom course work and pursue continuously all over the first three years until beginning of the advanced pharmacy practice experience. Considering this modification, the students will be able to undertake direct patient-care responsibility and get involved in patient-centered care early (17). The participants of our study not only were not completely aware of their professional role, but also they were dissatisfied from their profession. Chevalier *et al.* mentioned three roles for pharmacists including assessor, educator and problem solver, who acts as an information resource, liaison, interpreter and transition enabler (18). Rapport *et al*. indicated the general agreement of pharmacists that pharmacists-patients relationship is influenced by external demands on their role, especially patients demand (19) which is in agreement with our results. 

In addition to implementing pharmacy ethics principles in daily routine, the pharmacists should be aware of the situations in which they have conflict of interest to manage it properly. The results of our study indicate that the participants were not able to manage conflict of interest especially in relation to pharmaceutical industries in the form of accepting their ads to be presented in the pharmacy environment, etc; because of unawareness. In fact determined relationships with industries are ethically acceptable and non-preventable. The most important problem is management of conflict of interest in the way that does not have negative impact on patients health and well beings (20). As stated, the pharmacists of our study mostly were not sensitive about the issue. Therefore, increasing pharmacists awareness and sensitivity to conflict of interest is of major importance which helps proper management of the issue. In this regard teaching and guidelines as well as policies are considered as proper measures for management of conflict of interest (21). 

**Table 1:** Demographic data of study participants in phase 1.

**Participants**	**number**	**Age range (yr)**
Technical manager and stakeholder	6	35-49
Technical manager in community pharmacy	3	30-52
Technical manager in government/institutional pharmacy	3	31-44

**Table 2:** Participants experiences and ethical challenges

**Experiences** **and** **challenges**	**Reasons** **and** **causes**	**Outcome**
lack of proper pharmacists-patients relationship	Non awareness of both patients and pharmacists, time shortage, limited physical environment, no financial profit, type of pharmacy, patients noncompliance	Nonprofessional behavior, lack of patients records
Poor management of medication error	Lack of knowledge and education, no guideline, no clarification about pharmacists role in Iran Health System	Lack of trust between pharmacist, patients and physicians
Non-OTC drug dispensing without prescription	No guideline, no proper teaching, depends on pharmacists policy, no	higher income, patients satisfaction
Lack of inter and intra professional relationship	Lack of inter- and intra-professional trust, misconceptions	Nonprofessional behavior, lack of professional integrity
Lack of pharmacists awareness about their responsibilities	Lack of knowledge, no teaching,	No responsibility, lack of professional integrity
No collaboration with custodian organizations	No proper relationship with regulatory bodies, insufficient potency of custodian organizations	Disorganized activities
Lack of awareness about professional rules and regulations	No teaching in undergraduate period, no proper reference	Disorganized activities
Lack of proper support for internal productions	Improper quality of domestic productions	High requirement to imported drugs
Dissatisfaction from profession	No responsibility, lack of acceptance by society and health professionals, high dependence on financial profit, mismatch between responsibility and income, no need to provide scientific pharmaceutical care	No professional integrity
Financial problems	Low profit, high expenses, inappropriate pricing, influence of investors and nonprofessional stakeholders	Conflict between profession and business
Mismanagement in confronting with ads and offers of pharmaceutical companies	Financial problems	Participation in pharmaceutical industries propagation
no official retirement plan	No need to provide pharmaceutical care, lack of awareness about the probable harms	Dropping off the quality of health care services
Uneven drug distribution during shortage	No organized distribution system, jobbery, mismanagement, not considering justice	Unfair drug rationing and distribution
Conflict of interest	Lack of knowledge, no guideline	Not considering patients interests as the first priority, nonprofessional behavior

**Table 3:** Demographic data of study participants in phase 2.

**Participants**	**Number**	**Age range (yr)**
Residents of clinical pharmacy	7	28-32
Specialists in pharmaceutics	6	32-34

**Table 4:** Recommendations generated from FGDs.

Knowledge development	Raising pharmacists knowledge and awareness about pharmacy practice Raising pharmacists knowledge and awareness about professionalism Raising pharmacists knowledge and awareness about pharmacy ethics Revising pharmD educational system Updating pharmacists knowledge and periodical evaluation Nurturing professionals at the beginning
Skill advancement	Teaching communication skills Increasing pharmacists self-confidence
Service provision	Empowering pharmacists in ethical decision making Documentation of pharmaceutical care for each patient Paying enough attention to the subsidized drug and their prescription Considering a special place for pharmaceutical consultation Rating pharmacies based on their services Promoting quality of domestic products Modifying pharmacists and pharmacies appearance Drug pricing based on quality
Regulation adjustment	Changing regulations Compiling a guideline for medication error Defining pharmacists role in health care system Compilation of a guideline for conflict of interest Organizing
Management and audit	Monitoring financial turnover of all parts of pharmaceutical system Monitoring conflict of interest Preventing dumping in drug distribution system Prohibiting corruption Empowering FDO Potential assessment of all of the pharmaceutical companies and stakeholders Organizing pharmaceutical ads Supervision and management of conflict of interest Promoting integrity among pharmacists Introducing a proper feature of the profession by media, etc Solving financial problems Creating proportionality between health system and pharmaceutical system Special attention to the pharmaceutical industries Providing strong support from pharmaceutical system Raising pharmacists authorities in industries Empowering and supporting pharmaceutical associations Assisting in novelty in drug production Omitting nonprofessional investors from pharmaceutical system Promoting contribution of multinational industries Defining the real pharmaceutical care tariff based on the service provided

FDO= Food and Drug Organization of Ministry of Healt

Pharmacoeconomics deals with affordable and efficient use of pharmaceuticals (22) which has impact on resource allocation especially when the resources are limited. In Iran most of the health care services are provided by public-sector known as Ministry of Health (MOH) and government has a centralized role in this regard. Furthermore the Iranian pharmaceutical market is highly related to the pharmaceutical imports especially for high-tech patented drugs which are under tight regulation of MOH. The Iran pharmaceutical system is challenged by drug price which is related to the exchange rate but the MOH tries to keep it constant, therefore most of the stakeholders of the community pharmacies are complaining of low profit margins. They claim that such a low benefit cannot overcome their expenses and they have to consider the other strategies to make their pharmacies profitable. The main problem in this system is the strong relationship between economy and financial profit and professional practice which strikes the quality of pharmaceutical care. Williams proposed revision of perception of profession in pharmacy practice. According to his view considering profit as contrary to professionalism is false reasoning (23). Thus it seems that MOH has to restructure the policies in order to dissociate the pharmacy practice from financial challenges. 

Drug shortage is a global problem and is on rise universally. Several stakeholders are responsible. Drug shortage has negative impacts on hospitals, and community pharmacies and makes troubles for patients (24). Unfortunately there is no guideline on managing drug shortage for pharmacists nor has been no study conducted to evaluate the scope, causes and impact of drug shortage in Iran. So each pharmacist acts based on his/her evaluation of the condition. As the results of our study show, this way of encountering with drug shortage diminishes justice in resource allocation and affects quality of care. 

In addition as the pharmaceutical industry is the strength of the pharmaceutical system, it should be involved in health-related economic strategies instead of being passive. 

Medication error and its devastating effects are preventable by development of a systematic approach. In our country there is no guideline to help medication error management. Pharmacists are at the end of the treatment chain and because of lack of a systematic approach every professional acts based on his/her experiences. Unfortunately lack of systematic approach diminishes patients confidence and increase health care costs. There are several reasons for medication errors including lack of knowledge, substandard execution, or system deficiency (25). 

One of the FGDs was performed by attending clinical pharmacy residents because we believe that the ethical professional services traces back to clinical pharmacy. Actually clinical pharmacists are one group of pharmacy specialists who are directly facing with patients and also ethical challenges. So their views may be of important notice. Mort *et al*. observed that fulfillment of IPPE course by focusing on clinical pharmacy practice, and communication skills improves clinical behaviors (26). 

In addition as the pharmaceutical industry seems to be the strength of each pharmaceutical system, supporting and potentiating this part may have positive impact. 

Generally the FGDs participants proposed knowledge development, skill advancement, service provision, regulation adjustment and management and audit for improving patients-centered pharmaceutical services. 

Because of limited exposure of our pharmacy students to the concept of professionalism and pharmacy ethics, it seems that the fundamental knowledge should be introduced to them with the chance of exerting the key concepts in discussions and peer reviews. For this purpose several ways have been proposed. Some researchers as King *et al*. evaluated an online course with a public health focus as beneficial in increasing the knowledge and capability of the pharmacy students to get informed and involved in ethical, social, cultural and governmental issues in pharmacy practice (27). In addition some offer inclusion of simulated learning modules centered on practice as a useful teaching strategy on the skill and professionalism of the pharmacy students (28), while Horton *et al* proposed an elective course (29). 

While in some countries pharmacists are considered as one of the trustiest professionals from ethical point of view and the society relies on their specialized knowledge and professional commitment, still the Iranian pharmacists could not find their real position in the health care services. In order to raise the Iranian pharmacists professional position, modification of infra-structures and rules and regulations in addition to revising the pharmacy educational system are highly recommended. In this regard, each issue and its problems have to be clarified and its solution be proposed. Pointing at the pharmacy ethics education, we revised the pharmacy ethics curriculum and we hope that the new curriculum be helpful. 

## Conflict of interest

The authors declare that they had no conflict of interest.
